# The Treatment of Diaphyseal Fractures

**Published:** 1918-10

**Authors:** 


***The Treatment of Diaphyseal Fractures.*** By. J. DELMAS. *La Presse Médicale*, June 20, 1918.

The author does not agree with Leriche that fractures in a bullet wound with regular orifices of entry and exit may be regarded as closed fractures, and treated without primary operation. He has found such wounds liable to infection even when the orifices are punctiform, and he considers primary operation essential. There should be generous excision, removal of bone fragments or foreign bodies, and primary suture. Such wounds are often as dangerous as those due to shell fragments.

Comparing a series of fractures with small orifices which he treated without operation, and another similar series treated by thorough excision and ablation, the author came to the conclusion that although the non-operated cases caused less inconvenience and at first appeared to do better than those given thorough surgical treatment, they were in a much less satisfactory condition at the end of twenty-five days. In general, the bone was poorly consolidated, the soft parts contained detached bone fragments which might later prove to be foci of infection, and not infrequently there was actually a fistulous discharge from the exit orifice.

The writer, therefore, would vary the generally accepted principle of abstention in cases of fracture with punctiform orifices as follows :

1. If only the entry orifice is punctiform, radical surgical treatment is indicated.
2. If both entry and exit orifices are punctiform, but X-ray examination discloses a comminutive fracture, radical surgical cleansing is indicated.
3. If both orifices are clean and punctiform, and the tract regular and without bone fragments, abstention may be warranted, provided that a careful watch is kept against possible infection from bits of clothing.

